# Efficient *α*, *β*-motif finder for identification of phenotype-related functional modules

**DOI:** 10.1186/1471-2105-12-440

**Published:** 2011-11-11

**Authors:** Matthew C Schmidt, Andrea M Rocha, Kanchana Padmanabhan, Zhengzhang Chen, Kathleen Scott, James R Mihelcic, Nagiza F Samatova

**Affiliations:** 1 Department of Computer Science, North Carolina State University, Raleigh, 27695, USA; 2 Computer Science and Mathematics Division, Oak Ridge National Laboratory, Oak Ridge, 37831, USA; 3 Department of Civil and Environmental Engineering, University of South Florida, Tampa, 33620, USA; 4 Department of Integrative Biology, University of South Florida, Tampa, 33620, USA

## Abstract

**Background:**

Microbial communities in their natural environments exhibit phenotypes that can directly cause particular diseases, convert biomass or wastewater to energy, or degrade various environmental contaminants. Understanding how these communities realize specific phenotypic traits (e.g., carbon fixation, hydrogen production) is critical for addressing health, bioremediation, or bioenergy problems.

**Results:**

In this paper, we describe a graph-theoretical method for *in silico *prediction of the cellular subsystems that are related to the expression of a target phenotype. The proposed (*α*, *β*)-motif finder approach allows for identification of these phenotype-related subsystems that, in addition to metabolic subsystems, could include their regulators, sensors, transporters, and even uncharacterized proteins. By comparing dozens of genome-scale networks of functionally associated proteins, our method efficiently identifies those statistically significant functional modules that are in at least *α *networks of phenotype-expressing organisms but appear in no more than *β *networks of organisms that do not exhibit the target phenotype. It has been shown via various experiments that the enumerated modules are indeed related to phenotype-expression when tested with different target phenotypes like hydrogen production, motility, aerobic respiration, and acid-tolerance.

**Conclusion:**

Thus, we have proposed a methodology that can identify potential statistically significant phenotype-related functional modules. The functional module is modeled as an (*α*, *β*)-clique, where *α *and *β *are two criteria introduced in this work. We also propose a novel network model, called the two-typed, divided network. The new network model and the criteria make the problem tractable even while very large networks are being compared. The code can be downloaded from http://www.freescience.org/cs/ABClique/

## Background

Identifying and understanding cellular subsystems (or functional modules) responsible for the expression of a phenotype can assist genetic engineers with determining which genes to introduce or modify [1] in order to aid (or inhibit) the phenotype expression in an organism. Identification of such subsystems (or functional modules) is often performed as a computational search for specific network structures, or *network motifs*, in network models of biological data [2,3].

A type of network model, called *protein functional association network*, lends itself to the identification of functional modules. In protein functional association networks, a pair of vertices representing proteins is connected by an edge if the genes that encode these proteins are functionally associated, i.e, needed for the same function [4]. Genes required for the same function may co-occur in the same operon, co-express under similar conditions, or be involved in gene fusion events [4]. Evidence of these phenomena can be empirically observed and used to predict the functional association of two genes.

Functional modules, in which all pairs of proteins are functionally associated, can be modeled as maximal cliques in the context of protein functional association networks [5,6]. Maximal cliques have been recognized for: (a) finding biologically more relevant protein complexes, with more than 10% improvement in their functional homogeneity when compared to clusters [6]; and (b) reducing the noise in the data [7]. Among all maximal cliques, we are only interested in those that potentially help an organism express a particular phenotype, and this requires additional signals.

One signal that can be observed in biological networks is the evolutionary conservation of a functional module. A module that is phenotype-related is more likely to be conserved in phenotype-expressing organisms than phenotype-non-expressing organisms [8,9].

With the nave approach (or the brute-force method), identifying maximal cliques that are statistically biased towards being present in the networks of phenotype-expressing organisms requires at a minimum comparision of all maximal cliques across all the organismal networks considered. For any given network, the number of maximal cliques can be exponential in terms of the network size. Thus, a multi-way comparison for such exponential spaces is impractical.

**Figure 1:**
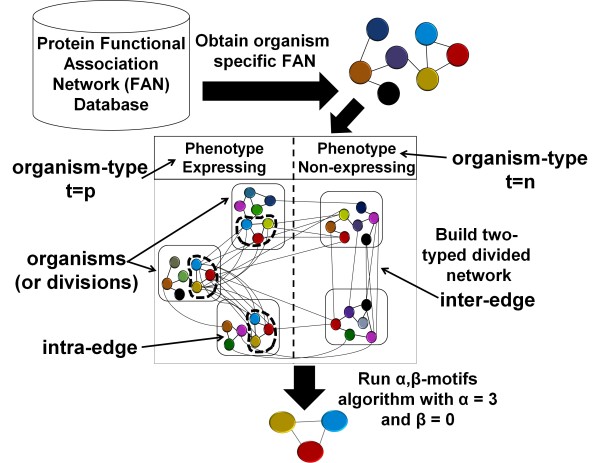
**Overview of the (*α*, *β*)-motif finder**.

**Figure 2:**
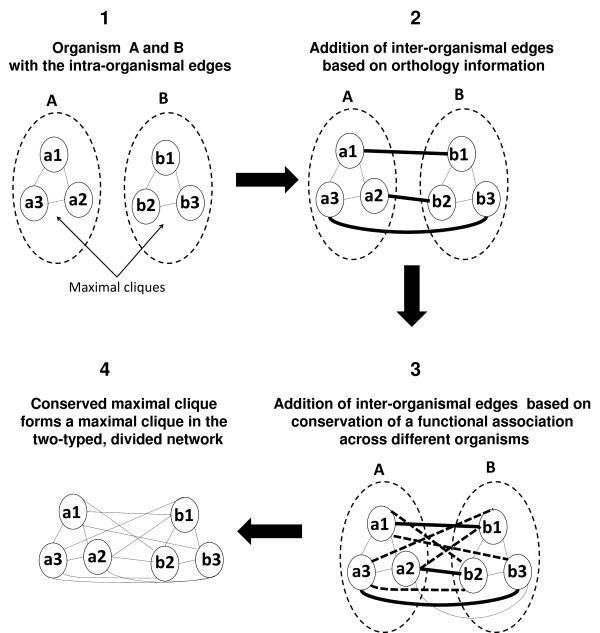
**A maximal clique conserved across multiple organismal networks forms a maximal clique in the two-typed, divided network**.

This paper describes an approach to make this problem tractable. The approach (Figure 1) introduces a novel network model called the *two-typed, divided network*. It then enumerates all of the maximal cliques in the constructed network that satisfy the introduced (*α*, *β*)-criteria. The maximal clique enumeration in the constructed network then translates to identification of the functional modules (modeled as a maximal clique) that are conserved across at least *α *phenotype expressing organisms and at most *β *phenotype non-expressing organisms, thus avoiding the need to perform multi-way clique comparisons (see Figure 2). It is hypothesized that this approach will move the problem of identifying the phenotype-related modules from the intractable space to the tractable space because the number of (*α*, *β*)-cliques will be much fewer than the total number of maximal cliques across all the organismal networks. The enumerated (*α*, *β*)-cliques are shown to be capable of modeling known phenotype-related functional modules (those found via literature search) through experiments with different target phenotypes.

## Results and Discussion

### Experimental Setup

Five experiments, each with a different target phenotype, were set up. The phenotypes used were hydrogen-production, acid tolerance, tricarboxylic acid (TCA) cycle expression, aerobic respiration, and motility (see Additional file 1). The organisms for the aerobic respiration and motility experiments come from Slonim *et al*. [10] and for all the other phenotypes, the organisms were identified through extensive literature search. Functional association networks for the organisms for all the experiments were downloaded from the STRING database [4]. Table 1 gives the statistics of the networks considered in the five experiments. In order to easily determine protein orthology, the proteins in an organism were replaced by their corresponding cluster of orthologous genes group (COGs [11]). COGs present in an organism were determined by information from the STRING database. Intra-organismal edges were defined by the functional association edges present in each organism. STRING database assigns a weight to each edge on the basis of the evidence that supports the functional association between the nodes at the two ends of the edges [4]. Hence, a threshold was applied to determine which intra-organismal edges were included in the network (Edge Threshold column in Table 1). The inter-organismal edges were defined in the manner described in the Methods section. In the STRING network, a threshold of above 700 is termed as "high confidence,'' so we only chose thresholds above 700.

**Table 1:** Phenotypes used to create the various networks used for experimentation

Network Name	Phenotype	Edge Threshold	Positive Organisms	Negative Organisms	Number of Vertices	Number of Intra-Edges	Number of Inter-Edges
HvnH_800	Hydrogen Production	800	9	8	23,397	270,681	2,752,378
TvrT_800	TCA Cycle Expression	800	14	6	25,433	300,025	3,481,638
AvAn_999	Aerobic Respiration	999	68	33	49,768	147,332	11,575,277
MvnM_999	Motility	999	85	56	72,272	198,028	23,743,770
ATvnAT_750	Acid Tolerance	750	8	5	17,535	227,296	1,888,286

In this section, the biological relevance of (*α*, *β*)-cliques is demonstrated by comparing the sets of genes predicted to be phenotype-related functional modules to known phenotype-related functional modules.

### Network Edge Threshold Selection

Our network edge threshold selection strategy aims to optimize the method performance on a small validation set of COGs that are known to be associated with the target phenotype. This prior knowledge is derived from published literature. Starting with the most conservative value (e.g., 999) of the edge threshold for the COG-COG organismal network in STRING database, we generate a set of networks till we reach a threshold of 500 with a 50 step difference. For each network, we calculate the accuracy of identifying known phenotype-related COGs from the validation set. We select the first threshold that ensures at least 75% accuracy. Additionally, we check the distribution of the number of unique COGs obtained per a given edge threshold and find the threshold that shows the maximum change in the number of COGs from the previous threshold and then analyze a few thresholds above and below that value using the prior knowledge information. See Additional file 2 for the details of the edge threshold experiment for the motility phenotype. The prior knowledge, or the validation set, for the motility phenotype was obtained from Liu *et al *[12] with *p*-value ≤ 0.05.

### Organism Selection

To identify phenotype-related functional modules for each of the 5 phenotypes (biohydrogen production, acid-tolerance, TCA expression, aerobic respiration, and motility), phylogenetically diverse (as much as possible) microorganisms representative of each phenotype were identified. Each phenotype was treated separately, and so the sets of organisms vary in each dataset (see Additional file 1). In some cases, for example, dark fermentative biohydrogen production and acid-tolerance, individual organisms were present across multiple datasets. Selection of organisms was based solely on the ability of each organism to express *at least one phenotype *discussed in the paper and not on the ability of each organism to express *all phenotypes *discussed in the paper. The organisms were chosen by reviewing the existing literature. The aerobic respiration and motility phenotypes were selected due to the availability of a number of completely sequenced and annotated genomes for the organisms exhibiting the phenotypes. In addition, to predict and distinguish between phenotypes that are highly similar, we selected the TCA expression phenotype.

In recent years, focus has shifted towards development of metabolically engineered organisms capable of expressing desired phenotypic traits. For the case of biohydrogen production using wastewater, this concept could be applied to create a mixed microbial community that is "ideal'' for enhancing hydrogen production. This is particularly important, since multiple phenotypes are necessary to optimize overall hydrogen yields, and not a single hydrogen-producing microorganism has been identified that is capable of expressing all these phenotypes. Thus, phenotypes important for hydrogen production using wastewater and waste materials were analyzed. Therefore, phenotypes selected for the study related to biohydrogen production using wastewater include:

• Hydrogen Production: In order to understand metabolic and cellular processes involved in expression and regulation of the hydrogen production phenotype, microorganisms, representative of all three types (dark fermentation, light fermentation, and bio-photolysis) of hydrogen production are selected to identify phenotype-related functional modules.

• Acid-tolerance (pH = 4.5-6.5): Acid-tolerant organisms are those capable of growing in slightly acidic and acidic conditions (pH 4-6). Similar to acidophiles, acid-tolerant organisms have developed metabolic and cellular acid tolerance response (ATR) systems to protect themselves when exposed to acid environments [13]. For hydrogen producers, the presence of ATR systems is extremely important, particularly, with respect to acidogenesis. During acidogenesis, organic acids (e.g., butyrate and acetate) are produced, thus lowering the pH level in the medium. In solventogenic organisms, such as *Clastridium acetobutylicum*, the change in pH results in a metabolic shift from acidogenesis to solventogenesis. As a result, the organism will stop producing acetate and butyrate and will generate solvents (e.g., acetone and butanol). To prevent metabolic shifts and maintain conversion of glucose (or other sugar compound) to hydrogen at maximum yields, organisms need to be able to tolerate acidic pH conditions.

For acid-tolerence phenotype, we selected a subset of species, since a diverse, large set of completely sequenced acid-tolerant microorganisms was not available at the time of the study. Prior to the experiment a number of acid-tolerant organisms were identified through literature review. However, many of the organisms' genome sequences were not completely sequenced. To ensure the best predictions, a criterion for organism selection was the presence of their sequenced and annotated genomes. Unfortunately, the organisms we identified are only representative of a small group of acid-tolerant bacteria consisting of nine Firmicutes and one Proteobacteria. As such, results obtained are somewhat biased towards acid-tolerant Firmicutes.

#### Hydrogen Production

The set of (*α*, *β*)-cliques was enumerated for three different statistically significant (*p*-value less than 0.005) *α*, *β*-values: (7,0) (see Additional file 3), (8,1) (see Additional file 4), and (9,2) (see Additional file 5). The enumerated (*α*, *β*)-cliques were able to identify sets of COGs that were known to be associated with hydrogen production.

Four types of COGs for maturation of [NiFe]-hydrogenase as present in hydrogen producing organisms and absent in hydrogen non-producing organisms were identified. Proteins associated with these COG groups are HypC (COG0298), HypD (COG0409), HypE (COG0309), and HypF (COG00068) (Table 2). In model organisms such as, *Escherichia coli*, HypCDEF proteins are described as regulators for maturation of uptake hydrogenase through participation in development of the active center [14,15]. Regulation is conducted through the requirement of insertion of Fe, Ni, and diatomic ligands by HypA-F proteins into the hydrogenase center for activation and maturation [16]. In this process, HypE and HypF are responsible for synthesis and insertion of Fe cyanide ligands into the hydrogenase's metal center. However, to carry out this process, HypC and HypD must form a complex for construction of the cyanide ligands to occur [15,17].

**Table 2:** A functional module identified by the (*α*, *β*)-motif finder for hydrogen production phenotype

COG ID	General Description	Gene name
COG0068	Hydrogenase maturation factor	hypF
COG0298	Hydrogenase maturation factor	hypC
COG0309	Hydrogenase maturation factor	hype
COG0409	Hydrogenase maturation factor	hypD

Based on published studies of crystal structures for hydrogenase maturation proteins, the presence and coordinated interaction between the proteins are essential for synthesis of [NiFe]-hydrogenase. In this study, we found similar evidence of functional associations between HypCDEF proteins. This is shown in one of the modules identified as associated with hydrogenase. While associations between maturation proteins have been well characterized in model organisms [15,18], detailed molecular analysis of [NiFe]-hydrogenase structures and their associated proteins has not been conducted across all phenotype-expressing organisms. Based on results obtained, it can be hypothesized that HypCDEF proteins are related to hydrogen producing organisms and will not be present in hydrogen non-producing organisms.

In addition to hydrogen maturation proteins, the (*α*, *β*)-motif finder was able to identify a module consisting of COG groups (COG1348 and COG 2710) whose functions were associated with expression of the nitrogen iron protein (NifH) and the molybdenum iron protein (NifD) (Table 3) [19]. Together these proteins comprise two essential components of nitrogenase, a key enzyme in nitrogen-fixation [20]. During nitrogen-fixation, nitrogenase catalyzes the conversion of nitrogen gas to ammonia and inadvertently results in the production of hydrogen gas as a byproduct [19,21]. To carry out this process, NifD serves as the binding site for substrates, while NifH assists in biosynthesis of co-factors for NifD [20]. While these proteins are associated with the nitrogen fixation phenotype, results from our algorithm suggest that these proteins are highly conserved across various hydrogen producing organisms, thus they may play an indirect role in hydrogen production.

**Table 3:** A functional module associated with nitrogenase formation identified by the (*α*, *β*)-motif finder

COG ID	General Description	Gene name
COG0388	Predicted amidohydrolase	unknown
COG0446	Uncharacterized NAD(FAD)-dependent dehydrogenase	HcaD
COG1063	Threonine dehydrogenase and related Zn-dependent dehdyrogenases	Tdh
COG1348	Nitrogenase subunit	NifH
COG2710	Nitrogenase molybdenum-iron protein	NifD

Although the presence of these two proteins is essential for nitrogen-fixation and biological hydrogen production, association of other genes may play an important role regulating Nif genes. Examples include proteins such as cysteine sulfinate desulfinase (COG1104; NifS) and nitrogen regulatory protein PII (COG0347; GlnK), which are involved in synthesis of the Fe-S cluster and regulation of proteins responsible for nitrogen metabolism [22], respectively. For both GlnK and NifS, the (*α*, *β*)-motif finder predicted associations between each COG group and Nif proteins. Specifically, we noted the association of NifH with the regulatory protein PII (GlnK). In nitrogen fixing organisms, GlnK is described as a key signal transducer in NifA in some organisms and regulatory protein in the transcription of the nitrogenase protein NifH in other organisms [23]. In this study, the association between COG groups related to NifH and GlnK supports experimental evidence that PII proteins are involved in inactivation of nitrogenase across a number of nitrogen-fixing species. In addition, identification of this COG-COG associations suggests that PII proteins may play a vital role in hydrogen production via nitrogenase.

#### Acid Tolerence

When a set of acid-tolerant microorganisms (Phylum Firmicutes and Proteobacteria) were used by the (*α*, *β*)-motif finder (see Additional file 6), the two main mechanisms, lysine/arginine decarboxylase and arginine deaminase, associated with acid-tolerance were not identified. However, eleven types of COGs associated with amino acid transporters were identified, suggesting that amino acid transport is highly related to the set of phenotype-expressing organisms in this study. Within microorganisms, amino acid transporters can participate in a number of metabolic and cellular processes, such as energy metabolism and protein synthesis. In organisms exposed to acid stress, decarboxylation of the two amino acids, lysine and arginine, is reported as two mechanisms for neutralization of internal pH [13,24,25]. During the neutralization process via arginine decarboxylation, antiporters responsible for replacing the argmatine generated from arginine with another arginine brought in from the surrounding environment [24]. In another system, arginine deaminase, ammonia is generated to help protect against acid stress [13]. From our knowledge of these systems, production or uptake of amino acids by microorganisms may play an important role in regulating intracellular pH levels.

In this study, eleven COG groups for amino acid transport were predicted as present across 10 acid-tolerant microorganisms. Proteins associated with these COG groups include argininosuccinate lyase (COB0165; ArgH) and the amino acid transporter LysP (COG0833) (Table 4). Argininosuccinate lyase is responsible for degrading argininosuccinate to form arginine and fumarate. LysP amino acid transporter is a permease system used by some microorganisms to transport lysine into cells [26]. Similar to arginine, decarboxylation of lysine has been linked to acid response by some bacteria [27]. While the transport of lysine by the LysP amino acid transporter system is not inhibited by arginine, arginine has been reported to regulate utilization of lysine by the lysine decarboxylation pathways [27]. While the direct interaction between lysine transport and lysine production is not clear, results suggest that there is some regulatory control occurring between these two systems.

**Table 4:** A Functional module identified by the *α*, *β*-motif finder for acid tolerance phenotype

COG ID	General Description	Gene name
COG0165	Argininosuccinate lyase	ArgH
COG0833	Amino acid transporter	LysP

#### TCA Cycle

The tricarboxylic acid (TCA) cycle is a metabolic pathway involving eight different enzymes related to energy production in aerobic organisms. Therefore, it could be modeled by (*α*, *β*)-cliques in both the experiment comparing TCA expressing organisms to reverse TCA (rTCA) expressing organisms (TvrT_800) and the experiment comparing aerobic to anaerobic organisms (AvAn_999). The set of COGs that contain enzymes known to be involved in the TCA cycle are given in Table 5. Since some of the enzymes involved in the TCA cycle are not specific to organisms that express the TCA cycle [28], comparative *in silico *methods have difficulty identifying all of the enzymes involved in the pathway.

**Table 5:** COGs associated with TCA cycle enzymes and whether any of them are part of an (*α*, *β*)-clique enumerated in either two-typed, divided network

Enzyme Name	Associated COG IDs	TvrT_800	AvAn_999
Citrate synthase	COG0372	Yes	Yes
Aconitase	COG1048, COG1049	Yes	Yes
Isocitrate dehydrogenase	COG0473, COG0538, COG2838, COG4579	Yes	No
2-oxoglutarate dehydrogenase	COG0022, COG0508, COG0567, COG1071, COG1249	Yes	Yes
Succinyl-CoA synthetase	COG0045, COG0074	Yes	Yes
Succinate dehydrogenase	COG0479, COG1053, COG2009, COG2142	Yes	Yes
Fumarase	COG0114	Yes	Yes
Malate dehydrogenases	COG0039	Yes	Yes

To determine if this functional module could be modeled as an (*α*, *β*)-clique, the set of (*α*, *β*)-cliques with significant *α*, *β*-values were enumerated in both networks. In the TvrT_800 network, this was defined as any *α*, *β*-value that had a *p*-value less than 0.005. In the AvAn_999, this was determined as any *α*, *β*-value that had a *p*-value less than 1 * 10^-5^.

The statistically significant (*α*, *β*)-cliques enumerated in the TvrT_800 network (see Additional file 7) contained COGs that represented all eight of the TCA enzymes, while those enumerated in the AvAn_999 network (see Additional file 8) contained COGs representing seven of the eight TCA enzymes. One of the (*α*, *β*)-cliques in the TvrT_800 network contained COGs representing seven of the eight enzymes in the TCA cycle (Table 6). It is significant to note that in the (*α*, *β*)-clique identified in Table 6, only one of the COGs has an individual distribution that is significantly biased towards TCA expressing organisms. In the AvAn_999 network, the COGs representing the TCA enzymes were less likely to be included in the same (*α*, *β*)-clique. This is likely due to the higher edge threshold used in this network.

**Table 6:** The set of COGs modeled by an (*α*, *β*)-clique enumerated in the TvrT_800 network.

COG ID	COG Description	Positive Organisms	Negative Organisms
COG0039	Malate/lactate dehydrogenases	12	6
COG0045	Succinyl-CoA synthetase, beta subunit	12	6
COG0074	Succinyl-CoA synthetase, alpha subunit	12	6
COG0372	Citrate synthase	14	6
COG0473	Isocitrate/isopropylmalate dehydrogenase	13	6
COG0479	Succinate dehydrogenase/fumarate reductase, Fe-S protein subunit	13	6
COG0567	2-oxoglutarate dehydrogenase complex, dehydrogenase (E1) component	12	0
COG1048	Aconitase A	12	2
COG1053	Succinate dehydrogenase/fumarate reductase, flavoprotein subunit	14	6
COG1249	Pyruvate/2-oxoglutarate dehydrogenase complex, dihydrolipoamide dehydrogenase (E3) component	13	4

The number of positive and negative organisms each COG is present in is given in the rightmost two columns. The complete (*α*, *β*)-clique is present in 11 positive organisms and 0 negative organisms.

#### Motility

The motility phenotype was examined to observe how both the (*α*, *β*)-clique model and the enumeration algorithm performed when a large number of organisms were used. The two-typed, divided network used in this experiment (MvnM_999) was constructed using 85 functional association networks from motile organisms and 56 functional association networks from non-motile organisms. The (*α*, *β*)-cliques were enumerated in the phylogenetic functional association network for all *α*, *β*-values that had a *p*-value of less than 1 * 10^-5^. This resulted in 95 unique (*α*, *β*)-cliques (see Additional file 9).

The enumerated set of (*α*, *β*)-cliques contained 38 (*α*, *β*)-cliques that consisted entirely of flagella-related proteins. Additionally, five (*α*, *β*)-cliques were enumerated that contained chemotaxis-related proteins. Flagella are cellular structures that enable the movement of microorganisms, while chemotaxis is a chemical process that determines how the microorganism moves in response to its environment [10]. These immediate results suggest that at least some of the remaining 52 (*α*, *β*)-cliques include COGs that are motility-related.

### Effects of Phylogenetic Diversity

In addition to introducing the phylogenetic diversity scoring function *S_p_*() (see Methods section), we performed an experimental study to test the robustness of our scoring method. For the hydrogen production phenotype, we grouped our input set of 17 organisms into 13 groups based on their *genus*. For each experiment, we randomly selected one organism from each genus, then chose a random subset (of size 8-13) of these organisms, and finally ran the (*α*, *β*)-motif finder algorithm on each subset. We then compared the results of these experiments and found that based on both the module's significance score and the module's phylogenetic score, the top 10 most significant modules identified remained unchanged. The organisms used in the experiments and the results for various experiments are avaliable in Additional files 10 and 11, respectively.

### Effect of (*α*, *β*)-criterion on the Number of Maximal Cliques

The results in this section describe the effect that different values of *α *and *β *have on the number of maximal cliques output when the algorithm is run on a given two-typed, divided network. The (*α*, *β*)-cliques corresponding to all possible values of *α *and *β *from two-typed, divided network of two phenotypes, hydrogen production and TCA cycle expression were enumerated (see Additional files 12 and 13) to analyze the effect.

**Figure 3:**
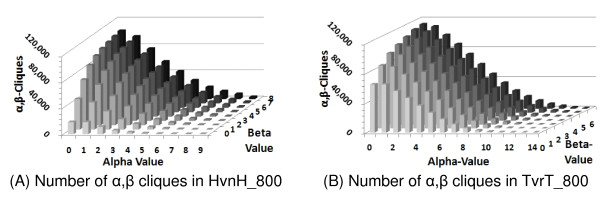
**The number of (*α*, *β*)-cliques for all ***α ***and ***β***-values in TCA cycle and hydrogen production two-typed, divided networks**.

Intuitively, the number of (*α*, *β*)-cliques decreased as the *α*-value increased and the *β*-value decreased. Thus, as shown in Figure 3, the maximum number of (*α*, *β*)-cliques occurs when the *α*-value equals zero and the *β*-value equals the number of negative divisions. This is because this criteria are equivalent to the enumeration of all the maximal cliques in the network. Alternatively, the minimum number of (*α*, *β*)-cliques get enumerated when the *α*-value equals the number of positive divisions and the *β*-value equals zero. Large *α *and small *β *is the problem space required for identifying (*α*, *β*)-cliques that may represent phenotype-related functional modules. Thus, as hypothesized, the introduction of the (*α*, *β*)-criterion moves the problem into a tractable space, because there are fewer (*α*, *β*)-cliques to enumerate for statistically significant values of *α *and *β*.

### Effect of Search Bounds on Algorithm Runtime

**Figure 4:**
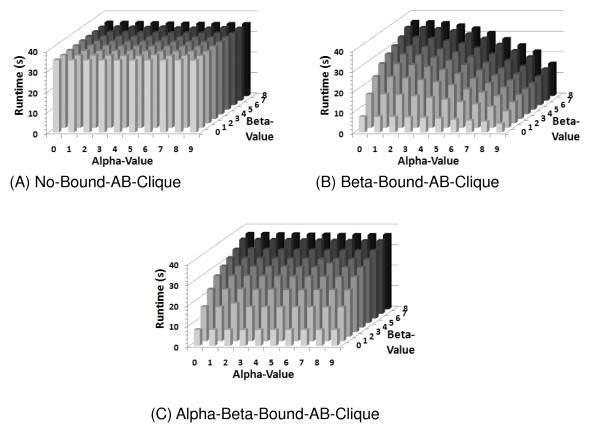
**The runtimes of the three implementations of Algorithm 1 for all ***α ***and ***β***-values in the HvnH_800 network**.

This section presents results on the effectiveness of two search bounds intended to reduce the search space and runtime of the algorithm as the number of (*α*, *β*)-cliques is reduced (see Additional files 12 and 13). Three implementations of Algorithm 1 were designed and their resulting runtimes were compared to demonstrate the effectiveness of the bounds. The first implementation, referred to as NO-BOUND-AB-CLIQUE, only used the output check on Line 2 of Algorithm 1 to ensure only (*α*, *β*)-cliques were enumerated. The second implementation, referred to as BETA-BOUND-AB-CLIQUE, added the bound on Line 7 of Algorithm 1 to bound the search space. The third algorithm, ALPHA-BETA-BOUND-AB-CLIQUE used all of the bounds in Algorithm 1 to bound the search space. Figure 4 demonstrates the effectiveness of the bounds for various values of *α *and *β *when enumerating (*α*, *β*)-cliques in the phylogenetic functional association network constructed from hydrogen-producing and hydrogen non-producing organisms (see Additional file 12). The lack of any variation in the runtimes of the NO-BOUND-AB-CLIQUE implementation for the various *α*, *β*-values is due to its lack of bounds (Figure 4A). The runtime of BETA-BOUND-AB-CLIQUE is also invariant as the *α*-value changes (Figure 4B). However, the reduction in its runtime correlates well with the reduction in the number of (*α*, *β*)-cliques as *β *decreases. This property holds for the ALPHA-BETA-BOUND-AB-CLIQUE implementation as well (Figure 4C). However, the reduction in the number of cliques as the *α*-value increases does not correlate as well with the reduction in the runtime of the ALPHA-BETA-BOUND-AB-CLIQUE implementation.

## Conclusion

In this paper, we proposed a methodology that can identify evolutionarily conserved functional modules that are likely associated with the expression of a target phenotype. The structure of the functional modules considered in the paper is a maximal clique. The task of enumerating the maximal cliques in a network and comparing maximal cliques across multiple networks to identify those biased towards phenotype-expressing organisms can be computationally intractable for large networks. In this paper, we have introduced a novel type of network model called the two-typed, divided network and the (*α*, *β*)-criterion that together help move the problem to a tractable space with the modules known to be related to the target phenotypes considered. Additional files 14, 15, 16, 17, 18, 19 and 20 provide additional information about the (*α*, *β*)-cliques identified for the five target phenotypes.

## Methods

### Overview

The following steps provide the pipeline of our methodology:

1. Build the two-typed, divided network model which involves combining the individual organismal networks into the single network.

2. Identify statistically significant *α *and *β *values using hypergeometric test.

3. Run the (*α*, *β*)-motif finder algorithm to identify the phenotype-related functional modules.

4. Assign a phylogenetic diversity score to each functional module identified in the previous step.

5. Run Hendrix *et al *[29] algorithm using the functional modules identified and each of the organismal networks used in the (*α *- *β*)-motif finder as input, to account for the missing edges in the organismal networks.

### Two-typed, Divided Network Model

In a *divided network N *= (*D*, *V*, *E*), the set of vertices *V *is completely divided amongst non-intersecting subsets of vertices, called *divisions*, which are represented by the set of subsets *D*. Edges in a divided network can be either *inter-organismal *edges or *intra-organismal *edges. If an edge connects two vertices that belong to different divisions, then the edge is considered an inter-organismal edge. If an edge connects two vertices that belong to the same organism, then the edge is considered an intra-organismal edge.

Given the functional association networks of *k *organisms, the divided network *N *with *k *divisions representing the *k *organisms is constructed. Each division includes all of the proteins from a single organism. Thus from hereon, a division will be simply referred to as an organism. An intra-organismal edge represents the functional association of the two proteins being connected by the edge (Figure 2.1). An inter-organismal edge can model two different relationships: (1) it can connect two proteins from different organisms that are functionally orthologous (Figure 2.2) (orthology is identified with the help of COG data) or (2) it can represent the conservation of a functional association across different organisms (Figure 2.3). For an example of the latter, let *a*_1 _and *a*_2 _represent proteins in organism *A*, and let *b*_1 _and *b*_2 _represent proteins in organism *B*. The edges (*a*_1_,*b*_2_) and (*a*_2_,*b*_1_) would exist (Figure 2.3) if and only if the following criteria are met:

1. *a*_1 _and *a*_2 _are functionally associated in *A *(Figure 2.1);

2. *b*_1 _and *b*_2 _are functionally associated in *B *(Figure 2.1);

3. *a*_1 _and *b*_1 _are orthologous proteins (Figure 2.2);

4. *a*_2 _and *b*_2 _are orthologous proteins (Figure 2.2);

The concept of a *organism-type *is introduced to differentiate the organisms according to the expression of the phenotype. Each organism will be associated with a type *t*. A phenotype-expressing organism will belong to the *positive type *(*t *= *p*) and a phenotype-non-expressing organism will belong to the *negative type *(*t *= *n*). Due to the binary nature of phenotype expression and non-expression, the network model is called a *two-typed, divided network*, *N_t_*. The set of organisms that have the same type *t *are referred to as a *type-set *of *t*, *T*(*t*). For a given clique *S *from *N_t _*and type *t*, a *type-count *function *c*(*S*, *t*) returns the number of organisms in the type-set *T*(*t*) that contain at least one vertex in *S*. This condition is sufficient to check if the conserved clique is present in an organism.

A clique in the two-typed, divided network (Figure 2.4) is made up of a set of cliques (e.g, the clique in Figure 2.4 is made up of two cliques of three vertices each: one clique from Organism A and the other clique from Organism B), where each individual organism contributes exactly one clique or nothing. Thus, the type-count function only needs to check if an organism is participating in the current two-typed, divided network clique or not. In other words, if any one vertex from the organism is present in a two-typed, divided network clique, then the organism contributes a clique. Thus, checking if at least one vertex of the clique is in the organism is sufficient.

### (*α*, *β*)-criterion

The (*α*, *β*)-criterion is introduced to reduce the number of maximal cliques that need to be compared across organismal networks and to identify those modules that are highly biased towards phenotype-expressing organisms. A subnetwork *S *satisfies the *α*-criterion, if the type count value *c*(*S*, *p*) ≥ *α*. A subnetwork *S *satisfies the *β*-criteria, if the type count value *c*(*S*, *n*) ≤ *β*. A subnetwork *S *satisfies the (*α*, *β*)-criterion if and only if it satisfies both the *α*-criterion and the *β*-criterion.

### Parameter Threshold Selection

Thresholds for statistically significant values of *α *and *β *parameters are identified using the hypergeometric test. Given a population *P *(i.e., the set of all input organisms), let *S *be the set of successes (i.e., all the phenotype expressing organisms) in the population, let *X *be a sample from *P *and *Y *be the set of successes in the sample *X*. The null hypothesis states that a random draw of *X *organisms from *P *with |*S*| successes will yield |*Y*| successes. The alternate hypothesis states that a random draw of *X *organisms from *P *with |*S*| successes will not yield |*Y*| successes.

If *P *is the set of all input organisms, *S *is the set of all phenotype-expressing organisms, and *S *- *P *is the set of all phenotype non-expressing organisms, then hypergeometric test provides the *p*-value for all *α *values from 1 to |*S*| in combination with all *β *values from 0 to |*S *- *P*|. For each test, the *α *+ *β *becomes the size of the sample *X *and the *α *value becomes *Y*, while *S *and *P *are the same as described earlier. We choose all (*α*, *β*) pairs with *α *≥ *β *that have *p*-value less than or equal to a specified threshold (e.g., *p*-value is less than 0.005).

Additionally, for each *β *value, we choose only the smallest *α *value such that the associated pair (*α*, *β*) satisfies the given threshold for its *p*-value. This way only *non-redundant *pairs are considered and (*α*, *β*)-cliques are identified if the maximal clique exists in **at least ***α *of positive networks. Thus, if a maximal clique exists for *α*_0_, then it is also part of the resulting set for *α*_0 _+ 1 for the same value of *β*. Thus, the smallest *α *value will contain all of the possible cliques for the given *β *value.

In the hydrogen production phenotype experiment, |*P*| = 17, |*S*| = 9 and |*S *- *p*| = 8 and a *p*-value threshold of 0.005, we identify all those non-redundant (*α*, *β*) pairs with *p*-value ≤ 0.005. Additional file 21 contains these results for the hydrogen production phenotype.

Note that this significance test does not take into account the phylogenetic similarity or phylogenetic diversity of the organisms in the input set and in the selected sample. It only provides the candidate (*α*, *β*) pairs used for running the algorithm. The additional scoring of the identified (*α*, *β*)-clique based on phylogenetic diversity of the organisms is further applied, as described below.

### Phenotype-related Functional Module Identification

If it is assumed that a functional module in a single organism will form a maximal clique in the organism's functional association network, then a functional module that is evolutionarily conserved will form a maximal clique in the two-typed, divided network (Figure 2). A functional module is present in two different organisms if there is a one-to-one mapping between the proteins of the module in one organism and the proteins of the module in the other organism. The one-to-one mapping is obtained by using orthology (Figure 2.2). By virtue of the existance of intra-organism edges and the addition of the inter-organismal edges (based on the two criteria discussed earlier), a conserved functional module, will form a maximal clique in the two-typed, divided network (Figure 2.4).

We hypothesize that conserved functional modules that are related to phenotype-expression will likely form maximal cliques and satisfy the (*α*, *β*)-criterion in the two-typed, divided network. Given a large enough *α*-value, the *α*-criterion will ensure that the maximal clique is present in enough phenotype-expressing organisms to likely represent a phenotype-related functional module. The *β*-criterion will ensure that the functional module is less likely related to the phenotype-non-expression. The maximal cliques that satisfy the (*α*, *β*)-criteria are referred to as (*α*, *β*)-cliques.

The main distinction to make here is that not every maximal clique in the two-typed, divided network is an (*α*, *β*)-clique. A maximal clique is also an (*α*, *β*)-clique if at least *α *of the phenotype-expressing organisms and no more than *β *phenotype non-expressing organisms participate in the maximal clique identified from the two-typed, divided network. Hence, as we enumerate the maximal cliques, we keep track of the the number of phenotype expressing and phenotype non-expressing organisms that participate in this clique and only output those that satisfy both the *α *and *β *criteria. A second distinction to make is that a maximal clique of the two-typed, divided network is not the relevant motif; instead, it is a set of maximal cliques (motifs), at most one from each organism. These maximal cliques are equivalent to each other and are representatives of a conserved phenotype-related motif.

### *α*, *β*-motif Finder

 **Input: **CLIQUE - The set of vertices in the current clique

 **Input: **CAND - The set of vertices that can be added to the set to form a new clique

 **Input: **NOT - The set of vertices that, if added to the set, would form redundant cliques

**1 if ***CAND is empty ***then**

**2**      **if ***NOT is empty AND c(CLIQUE, p) *≥ *α AND c(CLIQUE, n) *≤ *β ***then**

**3**         Output CLIQUE;

**4**      **return**

**5 **current = First vertex in CAND;

**6 while ***current *≠ *null ***do**

**7**      **if ***c(CLIQUE, n) *≤ *β ***then**

**8**         NEWCLIQUE = CLIQUE + current;

**9**         **forall ***vertices v in CAND ***do**

**10**            **if ***isConnected(v, current) ***then**

**11**               NEWCAND + = *v*;

**12**      **forall ***vertices u in NOT ***do**

**13**         **if ***isConnected(u, current) ***then**

**14**            NEWNOT + = *u*;

**15**         **if ***c(NEWCLIQUE *∪ *NEWCAND, p) *≥ *α ***then**

**16**            Call *α*, *β*-motif Finder(NEWCLIQUE, NEWCAND, NEWNOT);

**17**         CAND = CAND - current;

**18**         NOT = NOT + current;

**19**         **if ***CAND has more vertices ***then**

**20**            current = Next vertex in CAND;

**21**         **else**

**22**            current = null;

**Algorithm 1: **(*α*, *β*)-motif Finder Algorithm

The pseudocode of a recursive enumeration algorithm for (*α*, *β*)-motif finder is given in Algorithm 1. The algorithm is a modification of the maximal clique enumeration algorithm of Bron and Kerbosch [30], which will be referred to as the *BK *algorithm (see Additional file 22). There are two key modifications introduced to generate these phenotype-related modules. The first is the introduction of the type-count function *c*(*S*, *t*) dicussed in methods section. The function is used in Line 2 to restrict the output of the algorithm to (*α*, *β*)-cliques.

The second modification is the introduction of two bounds to reduce the search space and to make the algorithm more efficient. As the *BK *algorithm traverses the search tree it keeps track of three arrays. First is the CLIQUE array, which contains all of the vertices already in the clique. Second is the CAND array that keeps track of all of the vertices that could be added to CLIQUE to form a new larger clique. The third is the NOT array, which contains vertices that, if added to CLIQUE would only identify maximal cliques that have previously been enumerated. The first bound on Line 7 reduces the search space using the value of *c*(CLIQUE, *n*). If *c*(CLIQUE, *n*) is greater than *β*, then there is no reason to continue expanding the subtree of the current search node as the *β *criterion has already been violated. The second bound on Line 15 reduces the search space using the value of *c*(CLIQUE, *p*). For any given search node, the maximum *c*(CLIQUE, *n*) value that could exist for any child of the search node is *c*(NEWCLIQUE ∪ NEWCAND, *p*). If this value is less than *α*, then there is no reason for continuing the subtree expansion for the current search node.

### Statistical Significance of the (*α*, *β*)-cliques

Statistical significance of the (*α*, *β*)-clique being related to the phenotype-expression is quantified by calculating its bias towards the phenotype expressing organismal networks. This significance is calculated using hypergeometric probability, where the population is the total set of organisms in the experiment, the number of successes in the population is the number of phenotype expressing organisms in the experiment, the sample size is the total number of organisms that the (*α*, *β*)-clique is found in, and the successes in the sample is the number of phenotype expressing organisms the (*α*, *β*)-clique is found in.

### Phylogenetic Diversity Score of the (*α*, *β*)-cliques

In any comparative study, there is a possibility that the functional module is present across several networks purely due to phylogenetic similarity of the organisms. In order to quantify the phylogenetic diversity of the identified phylogenetically-related functional module, we introduce the *phylogenetic score *(*S_p_*()). This score takes into account the phylogenetic distance between all the organisms that the given module *M *is present in. Given the set *Org_P _*of phenotype expressing organisms that *M *is present in and the set *Org_N _*of phenotype non-expressing organisms that *M *is present in, the phylogenetic score is calculated as follows:

(1) $$S_{p}\left(M\right)=\frac{PP-PN-NN}{PP+PN+NN}$$

(2) $$PP=\underset{i\in Org_{P}}{\sum }\underset{j\in Org_{P}}{\sum }\delta \left(i,j\right)$$

(3) $$PN=\underset{i\in Org_{P}}{\sum }\underset{j\in Org_{N}}{\sum }\delta \left(i,j\right)$$

(4) $$NN=\underset{i\in Org_{N}}{\sum }\underset{j\in Org_{N}}{\sum }\delta \left(i,j\right)$$

where *δ*(*i*, *j*) is the phylogenetic distance between organism *i *and organism *j*. The function *S_p_*() rewards the module if the phylogenetic distance between the phenotype-expressing organisms is large, i.e, the module is conserved among a set of diverse phenotype-expressing organisms. The phylogenetic distance information was downloaded from the IMG (Integrated Microbial Genomes) database [31]. Section ''Effects of Phylogenetic Diversity'' and Additional file 23 provide the results of the phylogenetic score calculation and the robustness of the scoring function.

### Handling Missing Data in Input Graphs

With real data, it is quite possible to have missing information, for instance, unobserved protein interactions or missed orthologies, that would lead to missing edges and/or vertices in the network model of this data. Handling possibly missing edges becomes imporant, because the phenotype-related functional modules that we mine are modeled as maximal cliques. Hence, missed information in any one organism could lead to identification of cliques that do not model the complete phenotype-related sub-system.

To address this drawback, we introduce a post-processing step utilizing our quasi-clique mining algorithm with knowledge priors (DENSE) [29]. The algorithm takes as input a set of query vertices as knowledge priors and identifies those subgraphs that are "enriched'' by the vertices from the query set and meet a certain density (in terms of the number of edges) threshold. We supply all the identified phenotype-related modules as input to this algorithm and identify those extended functional modules that are more loosely connected, or form a quasi-clique. The density of the modules identified and the number of query vertices that will enrich the modules are controlled by the two parameters, *γ *and *μ*, respectively. The results of running the algorithm using the functional modules identified for the hydrogen production phenotype (*α *= 9, *β *= 2)and the functional association network of *Clostridium acetobutylicum *ATCC 824 with *μ *= 0.5 and *γ *= 0.5 can be found in Additional file 24.

Comparison of the unique COGs identified by the two algorithms (see Additional file 25) indicates that the DENSE algorithm was able to identify key hydrogen producing-related COGS missed by the (*α*, *β*)-motif finder algorithm. One example of a COG missed includes the iron only hydrogenase (COG4624). Enzyme complex associated with this COG plays a key role in the generation of biological hydrogen in microorganisms. In our initial results (see Section Hydrogen Production), the (*α*, *β*)-motif finder identified COGs involved in maturation and activation of [NiFe]-hydrogenases. While [NiFe]-hydrogenases are important for regulating removal of biological *H*_2_(g) in organisms, it is Fe-only hydrogenases that are responsible for the production of *H*_2_(g). As such, identification of motifs containing Fe-only hydrogenases is important for understanding how these genes interact with other genes and sub-networks. Other potentially important COGs included those indirectly associated with hydrogen production. These included COGs involved in the formation of acetate and Acetyl-CoA(COG0282, COG1013, COG1014). While these COGs do not lead to the direct formation of *H*_2_(g), they are involved in metabolic pathways that result in the production of *H*_2_(g). For example, COG0282 corresponds to acetate kinase, the last enzyme in acidogenesis. During the overall pathway for acidogenesis, hydrogen gas is produced. Although acetate kinase only catalyzes the conversion of acetyl-phosphate to acetate and not hydrogen, it is an indicator of acidogenesis. Hence, acetate production can be considered a phenotype-related pathway for hydrogen production.

### Time and Space Complexity of the (*α*, *β*)-motif Finder

The upper bound for the time complexity of the (*α*, *β*)-motif finder algorithm is the same as the worst case of the Bron and Kerbosh algorithm. The maximal clique enumeration problem depends on the number of cliques in the input graph, and in worst case that number can be $3^{\frac{n}{3}}$, where *n *is the number of vertices in the graph [32]. However, we have shown earlier [33] that biological networks typically do not exhibit this worst case behavior. Table 7 provides details on the density of all the networks used in the various experiments along with the time taken to enumerate all maximal cliques (not just those that satisfy the (*α*, *β*)-criterion). This is achieved by setting the the *α *value to zero and *β *value to the number of phenotype non-expressing organisms in the input two-typed, divided network. This criteria is the same as enumerating all the maximal cliques in the graph because any maximal clique in the graph will be present in at least 0 of the phenotype expressing organismal networks and can only be present in at most all of the phenotye non-expressing organismal networks. The runtime and memory usage for the various runs of the algorithm for the various phenotypes is included in Additional file 26.

**Table 7:** Runtime and Memory Usage in the Worst Case

Network	Density	Maximal Cliques	Maximal Cliques/sec	Total time (sec)	Memory Usage (MB)
HvnH_800	0.01105	103596	2851	36.34	23.41
TvrT_800	0.01169	113741	2218	51.29	29.23
MvnM_999	0.00917	11884	11	1093.78	186.21
AvAn_999	0.00139	13878	19	728.28	91.48
ATvnAT_750	0.01343	78395	2273	34.49	16.30

The memory usage by the algorithm, in the worst case, for the graphs used in the various experiments is documented in Table 7. As we discussed in [33], the Bron and Kerbosh algorithm, while exploring the graph in the depth-first manner, keeps track of the paths it has already visited and does not revisit them. Additionally, the algorithm uses a stack data structure, and hence the memory requirement is polynomial in the size of the input graph. However, the two-typed, divided network has more edges than the union of the edges across the organism-specific networks. The constructed network will have the same number of vertices, but it will have a superset of the edges in the union network. It will contain all of the edges in the union network, plus the inter-organismal edges, which do not exist in the union network. In the worst-case, the number of inter-organismal edges will be in *O*(*N*^2 ^* *k*), where *k *is the number of organisms and *N *is the number of nodes per organism-specific network. The alternative to adding these inter-organismal edges would be comparing the cliques enumerated in each organism-specific network with those enumerated in another organism-specific network. This would require *O*(*C^k^*) time, where *C *is the number of maximal cliques in an organism-specific network, which is likely significantly larger than *O*(*N*^2 ^* *k*).

## Authors' contributions

MS, KP and ZC developed and implemented the computational model and the algorithm and conducted computational experiments. AR and KS provided biological validation. MS, KP, and AR provided the initial draft of the manuscript. JM suggested and supervised the study related to the hydrogen production from wastewater and waste materials. NS provided the problem statement, supervised the developement of the computational methodology, and provided suggestions on methodology validation. JM, KS, and NF contributed to preparing the final version of the manuscript. All authors have read and approved the final manuscript.

## Supplementary Material

Additional file 1 **Supplement1-organisms**. List of organisms used in the various experiments. Click here for file

Additional file 2 **Supplements 28 Choosing Edge Threshold**. The experiment for choosing network edge threshold for motility experiment. Click here for file

Additional files 3 **Hydrogen production phenotype results for *α *= 7 and *β *= 0**. The (*α*, *β*)-cliques enumerated for the hydrogen production phenotype for *α *= 7 and *β *= 0. Click here for file

Additional files 4 **Hydrogen production phenotype results for *α *= 8 and *β *= 1**. The (*α*, *β*)-cliques enumerated for the hydrogen production phenotype for *α *= 8 and *β *= 1. Click here for file

Additional files 5 **Hydrogen production phenotype results for *α *= 9 and *β *= 2**. The (*α*, *β*)-cliques enumerated for the hydrogen production phenotype for *α *= 9 and *β *= 2. Click here for file

Additional file 6 **Acid-tolerance phenotype results**. The (*α*, *β*)-cliques enumerated for the acid-tolerance phenotype. Click here for file

Additional file 7 **Aerobic respiration phenotype results**. The (*α*, *β*)-cliques enumerated for the aerobic respiration phenotype. Click here for file

Additional file 8 **TCA cycle expression phenotype results**. The (*α*, *β*)-cliques enumerated for the TCA cycle expression phenotype. Click here for file

Additional file 9 **Motility phenotype results**. The (*α*, *β*)-cliques enumerated for the motility phenotype. Click here for file

Additional files 10 **Organisms used in Effects of Phylogenetic Diversity Experiments**. The list of organisms used in Effects of Phylogenetic Diversity Experiments. Click here for file

Additional files 11 **Effects of phylogenetic diversity**. Results of experiments conducted to test robusteness of our method along with the list of organisms used in each experiment. All experiments were conducted for hydrogen production phenotype. Click here for file

Additional file 12 **Algorithm runtimes for hydrogen production phenotype**. The (*α*, *β*)-motif finder algorithm runtimes for hydrogen production phenotype. Click here for file

Additional file 13 **Algorithm runtimes for TCA expression phenotype**. The (*α*, *β*)-motif finder algorithm runtimes for TCA expression phenotype. Click here for file

Additional file 14 **Size of (*α*, *β*)-cliques enumerated for aerobic respiration**. The size of (*α*, *β*)-cliques enumerated for aerobic respiration and the corresponding list of organisms they are present in. Click here for file

Additional files 15 **Size of (*α*, *β*)-cliques enumerated for hydrogen production (*α *= 7, *β *= 0)**. The size of (*α*, *β*)-cliques enumerated for hydrogen production (*α *= 7, *β *= 0) and the corresponding list of organisms they are present in. Click here for file

Additional files 16 **Size of (*α*, *β*)-cliques enumerated for hydrogen production (*α *= 8, *β *= 1)**. The size of (*α*, *β*)-cliques enumerated for hydrogen production (*α *= 8, *β *= 1) and the corresponding list of organisms they are present in. Click here for file

Additional files 17 **Size of (*α*, *β*)-cliques enumerated for hydrogen production (*α *= 9, *β *= 2)**. The size of (*α*, *β*)-cliques enumerated for hydrogen production (*α *= 9, *β *= 2) and the corresponding list of organisms they are present in. Click here for file

Additional file 18 **Size of (*α*, *β*)-cliques enumerated for motility phenotype**. The size of (*α*, *β*)-cliques enumerated for motility and the corresponding list of organisms they are present in. Click here for file

Additional file 19 **Size of (*α*, *β*)-cliques enumerated for tca cycle phenotype**. The size of (*α*, *β*)-cliques enumerated for tca cycle expression and the corresponding list of organisms they are present in. Click here for file

Additional file 20 **Size of (*α*, *β*)-cliques enumerated for acid tolerence phenotype**. The size of (*α*, *β*)-cliques enumerated for acid tolerence expression and the corresponding list of organisms they are present in. Click here for file

Additional file 21 **Selection of (*α*, *β*) thresholds for hydrogen production phenotype**. The selection of the set of (*α*, *β*) thresholds for the hydrogen production phenotype experiment. Click here for file

Additional file 22 **BK Algorithm**. Details of the Bron and Kerbosch [30] algorithm. Click here for file

Additional file 23 **Phylogenetic diversity score calculation**. Phylogenetic diversity score calculated for (*α*, *β*)-cliques enumerated for the hydrogen production phenotype. Click here for file

Additional file 24 **Extended functional modules obtained by running DENSE **[29]. The extended functional modules obtained by running Hendrix *et al *(DENSE) [29] using the phenotype-related functional modules obtained for hydrogen production phenotype as query vertices and *Clostridium acetobutylicum *ATCC 824 protein functional association network. Click here for file

Additional file 25 **DENSE vs. *α*, *β*-motif finder**. The comparison of unique COGs found by DENSE and *α*, *β*-motif finder. Click here for file

Additional files 26 **Runtimes and memory usage of various runs**. The runtimes and memory usage for the various runs of the various phenotypes. Click here for file
