# Supplementary material for: Efficient α, β-motif finder for identification of phenotype-related functional modules
Source: BMC Bioinformatics. 2011 Nov 11;12:440. doi: 10.1186/1471-2105-12-440 (PMC3287386; doi:10.1186/1471-2105-12-440)
Supplement: Additional file 22 — BK Algorithm. Details of the Bron and Kerbosch [30] algorithm. [file 1471-2105-12-440-S22.PDF]

Bron Kerbosch Algorithm (BK)

The BK algorithm [1] uses the recursive backtracking paradigm to enumerate all maximal cliques in the graph. At any given point in time it maintains three lists, C, I and X. The set C contains the vertices of the clique currently being enumerated, the set I contains vertices that are connected to all vertices in C and can be added to C to make a larger clique and the set X contains vertices that are connected to all vertices in C but are excluded from being added to C because all cliques containing vertices in X have already been enumrated in a different recusion cycle. Algorithm 1 gives an overview of the algorithm.

Algorithm 1: Bron and Kerbosch Algorithm

Algorithm: BK Algorithm
Input: A unweighted undirected graph $G = (V, E)$
Output: A list of all maximal cliques in G
$C = \emptyset$;
`/* A set of vertices that represent a maximal clique or can be extended to a maximal clique */`
$I = V(G)$;
`/* The set of vertices that are connected to all vertices in` C `and can be added to` C `to make a larger clique */`
$X = \emptyset$;
`/* The set of vertices connected to all vertices in` C `but excluded from being added to` C `*/`
$BK - Enumerate(C, I, X)$

Algorithm 2: The recursive function utilized in the **BK** algorithm

Algorithm: $BK - Enumerate(C, I, X)$
if $I = \emptyset$ *and* $X = \emptyset$ **then**
  print C as maximal clique;
else
  v = vertex connected to maximum number of vertices in I;
  while $v \neq \emptyset$ **do**
    $BK - Enumerate(C \cup \{v\}, I \cap N(v), X \cap N(v))$;
    `/*` $N(v)$ `represents the neighbors of vertex` v `in` G `*/`
    $I = P - \{v\}$;
    $X = N \cup \{v\}$;
    v = vertex connected to maximum number of vertices in I

The current recursion stops when C cannot be expanded any further, i.e., I becomes $\emptyset$. At this point, if set X is also $\emptyset$ then the vertices C form a maximal clique and added to the output set. The condition $X = \emptyset$ checks for maximality

because is X were not empty then it would mean the C can be further expanded with the vertices from X to form an even larger clique and hence C cannot be maximal.

References

[1] Bron C, Kerbosch J: **Algorithm 457: Finding All Cliques of an Undirected Graph**. *Commun ACM.* 1973, **16**(9):575577.
